# The COVID‐19 Pandemic and Its Impact on Children in Domestic Violence Refuges

**DOI:** 10.1002/car.2650

**Published:** 2020-08-18

**Authors:** Carolina Øverlien

**Affiliations:** ^1^ Norwegian Center for Violence and Traumatic Stress Studies (NKVTS) Oslo Norway; ^2^ Department of Social Work, Stockholm University Stockholm Sweden

**Keywords:** Domestic violence, Domestic abuse, Children, Wellbeing, Norway

## Abstract

- The COVID‐19 pandemic has resulted in negative consequences for children exposed to violence and abuse.
- Domestic violence refuge staff were greatly concerned about children both living outside and inside refuges.
- Domestic violence refuges have played a pivotal role during the COVID‐19 pandemic and should receive wider acknowledgement and greater support for their work.

## Introduction

In response to the rapid worldwide spread of the coronavirus, a national lockdown was announced in Norway on 12 March 2020. All daycare centres and schools were closed immediately, as were many small businesses, including restaurants and shops. Moreover, all gatherings, such as sporting events and concerts, were banned. These and numerous other restrictions in Norway during the spring of 2020 represented the most extreme measures enforced by the Norwegian government since the Second World War. Many of the restrictions have significantly impacted the lives of children and adolescents, especially the closing of daycare centres, schools and arenas in which many children spend their leisure time, such as football fields, swimming pools and art centres. Children and adolescents were not allowed to play with more than a few children at the same time – and preferably the same children each time – keeping at least one‐metre distance between them. Services vital for children and adolescents' physical and mental health, such as school health services and community health clinics for children and adolescents, were also closed or had reduced hours. In addition, child protection services in Norway reported reduced capacity due to employees' self‐isolation efforts, and that they were receiving fewer reports of abuse and neglect of children (Muladal, 2020).

Although the strict guidelines and rules have most certainly had an impact on all children and adolescents, children living in households with domestic violence may be at particular risk as a result of the pandemic. In the past few months, mainstream media, non‐governmental organisations (NGOs), international organisations and researchers have all called attention to the issue of child abuse and neglect as a particularly problematic consequence of social distancing and other virus control measures. End Violence Against Children (2020) emphasises the vulnerabilities of children during times of crisis, such as the COVID‐19 pandemic. A national helpline for children in Norway has reported a sharp increase in the number of calls from worried children since the lockdown, many concerning conflicts and tension at home (Jørnholt and Mjaaland, 2020). The World Health Organization (2020a) has reported an increase in violence and abuse against women and girls, and that security, health and financial concerns may increase tension in the home. A recently published overview of studies on the impact of pandemics on violence against women and children (Peterman *et al*., 2020) argues that ‘the regional or global nature and associated fear and uncertainty of pandemics provide an enabling environment that may exacerbate or spark diverse forms of violence against women and children’ (p. 3). Peterman *et al*. (2020) document nine direct and indirect causes of the link between pandemics and violence against women and children, among which are: economic instability; an inability for women (and children) to escape the abuser; and quarantine and social isolation. Families with low socioeconomic status and/or living in overcrowded areas are particularly vulnerable (Marques *et al*., 2020).

‘Children living in households with domestic violence may be at particular risk as a result of the pandemic’

Other voices – such as Green (2020), Bradbury‐Jones and Isham (2020) and Usher *et al*. (2020) – have also raised serious concerns about the impact of the COVID‐19 pandemic on children and adolescents. Green (2020) argues that, if attention is not shifted to the wellbeing of children exposed to violence and abuse, we risk an ‘irreversible scarring of a generation,’ as we might have a ‘second pandemic’ in the form of increased child abuse and neglect (p. 1). Bradbury‐Jones and Isham (2020) draw attention to the paradox that governments around the world have asked their citizens to stay home, but little attention has been paid to the consequences of this request for women and children living with domestic violence and abuse. Along these lines, Usher *et al*. (2020) call for governments and policy makers to take responsibility for raising awareness around the risk for increased domestic violence and abuse, and to spread information to the public about services and the need to report any abuse‐related concerns to the authorities. These editorials, published in international peer‐reviewed journals, emphasise the need for more research and knowledge building on all issues related to the pandemic and domestic violence. However, to date, there is very little empirical data on the impact of the pandemic on the issue of domestic violence and abuse, also from the perspective of service providers. In this short report, I provide insight into the situation of domestic violence refuges in Norway during the spring of 2020 and their concern for their youngest clients.

‘There is very little empirical data on the impact of the pandemic on the issue of domestic violence and abuse’

### Domestic Violence Refuges in Norway

Domestic violence refuges have existed in Norway since 1979. Refuges were originally meant for women and children; however, in 2010, men were also allowed to seek support and shelter in refuges (for a discussion on the inclusion of males in refuges, see Côté *et al*. (2018)). For the past 40 years, these refuges have been a haven in which victims of domestic violence and abuse can be safe from their abusive partners and parents; in addition, the refuges have also had the important role of putting domestic violence on the political agenda (Øverlien, 2011). Since the implementation of the Shelter Act in 2010, it is obligatory for all municipalities in Norway to provide refuge services to their inhabitants. With this new law, the local authorities became responsible for the total cost of refuge services in the country, and legal requirements regarding the content and quality of the services were imposed (Bakketeig *et al*., 2014). In this Act, children's rights are specified in accordance with the United Nations Convention on the Rights of the Child. This means that children and adolescents living in refuges with a parent have the right to have their needs for care, safety and schooling met, and be included in any issues that concern them and their everyday life at the refuge.

Many of the 46 domestic violence refuges in Norway have at least as many children as adults under their roofs. In 2018, 1842 adults stayed one or more nights at a refuge in Norway, and during the same year, 1452 children and adolescents under the age of 18 stayed with one of their parents at a refuge (The Norwegian Directorate for Children, Youth and Family Affairs, 2020). The length of stay varies greatly. Although the refuges are meant to be used for acute situations and short‐term protection of victims of violence and abuse, children often stay for long periods of time, together with a parent – especially in larger cities where there is a shortage of available and affordable housing.

## Aim and Methods

As both political driving forces and safe havens for victims of domestic violence, the refuges play a key role in Norwegian society. As such, when reports began arriving from different parts of the world concerning a possible increase of domestic violence and abuse during the COVID‐19 pandemic (see, for example, Godin, 2020), my colleagues and I constructed a web‐based survey to distribute to all refuges in Norway (*N* = 46). The survey was anonymous, the respondents were not asked any personal information about themselves, the refuge, or the clients. Hence, formal ethical approval from an ethical board was not required. The survey was distributed on 8 April 2020 with the aim of obtaining an overview of the impact of the COVID‐19 crisis and the virus control measures implemented on 13 March 2020 on victims of domestic violence and abuse.

The survey contained 15 items with fixed answers, follow‐up questions and an opportunity for the refuges to provide supplementary qualitative descriptions and examples. One reminder was sent on 20 April 2020 and the survey was closed on 23 April 2020. In total, 46 refuges took the survey, giving a response rate of 100 per cent. We asked the leaders of the refuges to respond. If they were unable, they could pass the survey on to an employee with extensive knowledge of the situation at the refuge. As the survey was taken between 8 and 23 April 2020, it covers the four to six weeks following the implementation of the government's strict, wide‐ranging virus control measures. The questions focused on four main topics: 1) changes in the services due to the pandemic; 2) the refuges' cooperation with other services; 3) what the refuge staff saw as most worrying in the current situation and what they saw as vital in order to support victims; and 4) changes in the requests and motivations for contacting the refuges. Some of the questions related specifically to children. The results of these questions, using descriptive statistics analysed in SPSS, are presented below. For additional insight into the concerns of refuge staff, everything from the comment field concerning children was also analysed.

‘[The survery] covers the four to six weeks following the implementation of the government's strict, wide‐ranging virus control measures’

## Results

As 56 per cent of the respondents reported that there had been a reduction in the number of requests from clients, many expressed concern that it is ‘too quiet out there,’ and that victims of domestic violence and abuse are not receiving the help and protection they need during the pandemic. One key survey question concerned specific groups that the refuge staff were particularly worried about: ‘During the coronavirus crisis, are there any groups of victims of violence and abuse that you are particularly worried are not getting the protection they need?’ The group the refuge staff reported being most concerned about was children, with a total of 83 per cent reporting this concern. As most children and adolescents were present in the home during the pandemic, and as many parents were working from home, parents and children were spending the majority of the day in the same physical space. Survey comments indicate that the refuge staff were concerned about children outside of the refuge living in households with domestic violence *and* children living in the refuges but not receiving the support that they need due to the pandemic.

‘Many expressed concern that it is ‘too quiet out there’’

### Concern for Children Living With Domestic Violence Outside of the Refuge

Regarding children living in households outside of the refuge and experiencing domestic violence, the refuge staff expressed great concern for children and adolescents living with abusive parents who may be experiencing increased stress during the pandemic. Almost half of the respondents (43%) reported that their clients believed the presence of children in the home during the day increased the risk of violence and abuse. In addition, 57 per cent answered that their clients reported that the virus control measures, and the coronavirus crisis itself, were increasing stress for the abusers. As one respondent commented:

‘We believe that children who lived with violence before the pandemic are now even more exposed … and that the exposed parent is now even less able to protect the child.’

And according to another:

‘With families living with prolonged stress, perhaps a worse economy and declining hopes for the future, it is easy to imagine that those who already struggle, now struggle even more. Our greatest fear is that we will see more suicides or familicide. If the hole gets too black and the road too long …’

In addition, the refuge staff were concerned that children exposed to violence who were living outside of the refuge would no longer receive the help that they need from professionals; and, moreover, that the violence they experience would remain undisclosed, as contact between adults outside of the family and the abused child is dramatically reduced. As one respondent reported:

‘We are concerned about the children who now have no support from the social services, at the same time as they are struggling because of isolation [not seeing their] friends and a closed‐down society – schools, daycare, after‐school activities, etc.’

Another expressed concern that:

‘Vulnerable groups, previously followed up with by different parts of the social and health sector, now have less contact and follow‐up, hence, fewer are “keeping track.” This also includes the children.’

Closed schools and daycare centres are of particular concern for the refuge staff as, for many children living with domestic violence and abuse, school represents normality and a zone free from the abusive parent. School staff can also be important in terms of supporting children living in difficult situations. In Norway, it is mandatory that school staff report concerns about abuse to the child protection services; as such, they also represent an important safety net for children and adolescents exposed to violence.

‘Closed schools and daycare centres are of particular concern for the refuge staff’

### Concern for Children and Adolescents Living at the Refuge

Many refuges in Norway cooperate with NGOs, such as Save the Children and the Red Cross, whose volunteers arrange activities for the children and help them with homework. Other refuges have rooms where the children can play with visiting friends. Some also cooperate with daycare centres and schools that send teachers to instruct those children living for longer periods at the centres and who are unable, for security reasons, to leave the refuge. However, social and pedagogical contacts such as these have been discontinued during the pandemic. One respondent explained that:

‘All activities for children arranged by volunteers, such as playgroups and help with homework, have ended … we no longer permit visitors from outside of the refuge.’

Another described it as follows:

‘All activities involving physical contact have stopped. All children are usually offered some kind of pedagogical activity at the centre, by schools or daycare centres, but that is now cancelled.’

According to a third respondent:

‘It has been really demanding with all the children who have not gone to school, but stayed at the refuge 24/7 with no alternative activities. Digital schooling and support from parents in crisis. We need more resources for those kids.’

‘Social and pedagogical contacts … have been discontinued during the pandemic’

Refuge staff also described how everyday life at the refuges has changed for the children since the start of the pandemic. In addition to the discontinuation of visits from friends, professionals and volunteers, the play areas, group meals and visits to other families' rooms have also been restricted. As one respondent wrote:

‘We ask everyone to spread out in the house, as much as possible, the children only play within their family, they can't sit with other children and eat, watching TV etc. All common meals at the centre have stopped … they can't visit each other's rooms. We have to watch that they don't get physically too close.’

### Services that are Flexible and Accustomed to Crisis Situation

While raising serious concerns, in particular about children and adolescents, the respondents' comments also underlined that refuges are accustomed to crisis situations, and that their staff members are flexible and creative and used to solving problems quickly. As one respondent stated:

‘We are educated, we keep calm, and we are highly trained to deal with challenging situations. Our staff has stepped up in every possible way.’

Another emphasised that ‘[o]ur strength is that we are good at readjusting and making the best of what we've got. We quickly got new routines in place.’

The pandemic has undoubtedly placed great demands on the refuges. As many as 90 per cent reported that they have had to adjust their organisation to accommodate the government's new requirements while simultaneously fulfilling their obligation to provide support and shelter for victims of domestic violence and abuse. However, the many comments about the situation faced by children, in particular, highlight that, though challenging, these adjustments include making changes with the best interest of the child in mind.

## Discussion

National strategies to the COVID‐19 pandemic differ between counties, and the re‐opening of societies is at different stages (World Health Organization, 2020b). In the neighbouring country Sweden, restrictions have not been as rigorously enforced as in Norway. At the time of writing, restrictions that have been lifted in Norway still remain in place in Ireland and the UK. Since the distribution of the survey reported on in this short report, daycare centres and schools in Norway have slowly begun to reopen. Some after‐school activities have also started up once more, and children and adolescents are again allowed to play football (albeit in smaller groups) on Oslo's public football fields. Some of the strictest virus control measures are being lifted, and the domestic violence refuges are reporting an increase in contact with clients, including children.

However, the lives of children and adolescents remain very much affected by the pandemic, and there is reason to believe that the last few months have seen an increase in violence against children. It is now of greatest importance that all social and health services, as well as daycare centres and schools, are vigilant concerning the negative consequences of the pandemic, now that children are returning after many weeks of self‐isolation. Norway has an extensive social and health sector focusing on children, and the issue of domestic violence and abuse has been high on the political agenda for several years. However, we know that violence towards and abuse of children too often remain undisclosed, and that many children do not get the support that they need and to which they have a right. Institutions and services for children and adolescents – particularly those who provide safety and support to children living with domestic violence and abuse – are now more crucial than ever. In addition to receiving the necessary support so that they can provide the same services as before the pandemic, they need additional resourses to be proactive, and find new, creative ways to reach out to children and adolescents in vulnerable situations. This includes domestic violence refuges, which have played a pivotal role during the COVID‐19 pandemic and will continue to do so. They need to be acknowledged for the work that they do and provided with the resources they need to not only keep children safe, but also support their schooling, arrange activities and allow for play, while continuing to follow the required social distancing and virus control measures.

‘There is reason to believe that the last few months have seen an increase in violence against children’
